# Supplementary material for: CXCR6+ natural killer cell immunotherapy preserves CD4+ T helper cells in humanized mice
Source: Front Immunol. 2026 Aug 10;17:1888755. doi: 10.3389/fimmu.2026.1888755 (PMC13501908; doi:10.3389/fimmu.2026.1888755)
Supplement: Supplementary file 1 [file DataSheet1.pdf]

Supplementary Material

1 Supplementary Tables

Supplementary table 1. Flow cytometry Abs staining

**Flowcytometry markers**	**Fluorophores**	**Clones**
hCD45	BV605	HI30
mCD45	AF532	30-F11
CD56	BV570	HCD56
DNAM-1	BUV563	DX11
CD57	PacBlue	HCD57
CD16	APC-Fire750	3GB
LILRB1	PE-Cy7	HP-F1
NKp46	BV650	9-E-2
NKG2D	BUV737	1D11
NKG2C	BUV615	134591
2B4	BV421	2-69
NKp30	BUV805	P30-15
KIR3DL1	PerCP-Cy5.5	DX-9
KIR2DL1	AF488	143211
KIR2DL3	AF700	180701
NKG2A	APC	Z199
CXCR6	PE/Dazzle 594	K041E5
CD69	BUV496	FN50
CD107a	BUV395	H4A3
CD3	APC/Fire	SK7
Granzyme B	BV510	GB11
Perforin	BV711	Dg9
IFN-y	BV750	4S.B3
T-bet	BV786	4B10
Ki67	BV480	B56
EOMES	PerCp-eflour 710	WD1928
CXCR6	BUV737	RUO
mCD45	Pacific Blue	30-F11
CD4	BV750	RU0
HIV Gag p24	FITC	Kc57-FITC
DAPI Live/Dead	Sigma Aldrich	

Gating Strategy and FMOs for human PBMCs

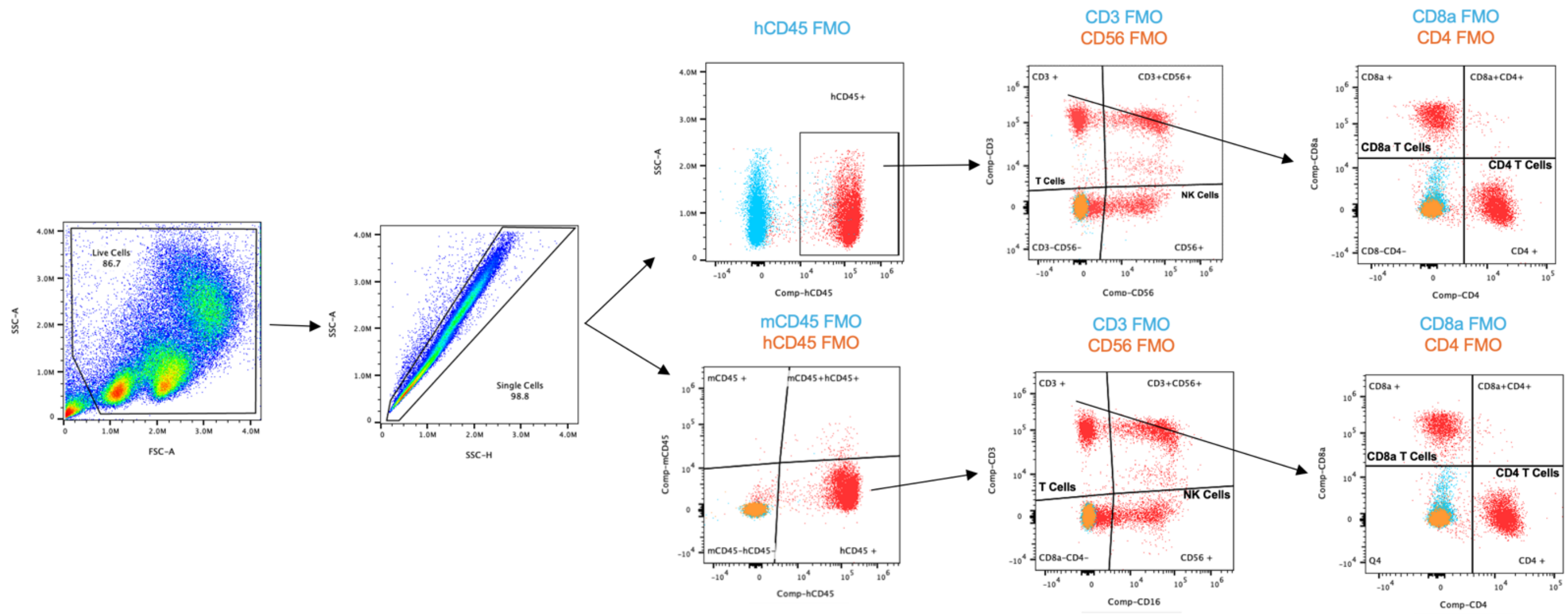

Gating Strategy and FMOs for humanized mice

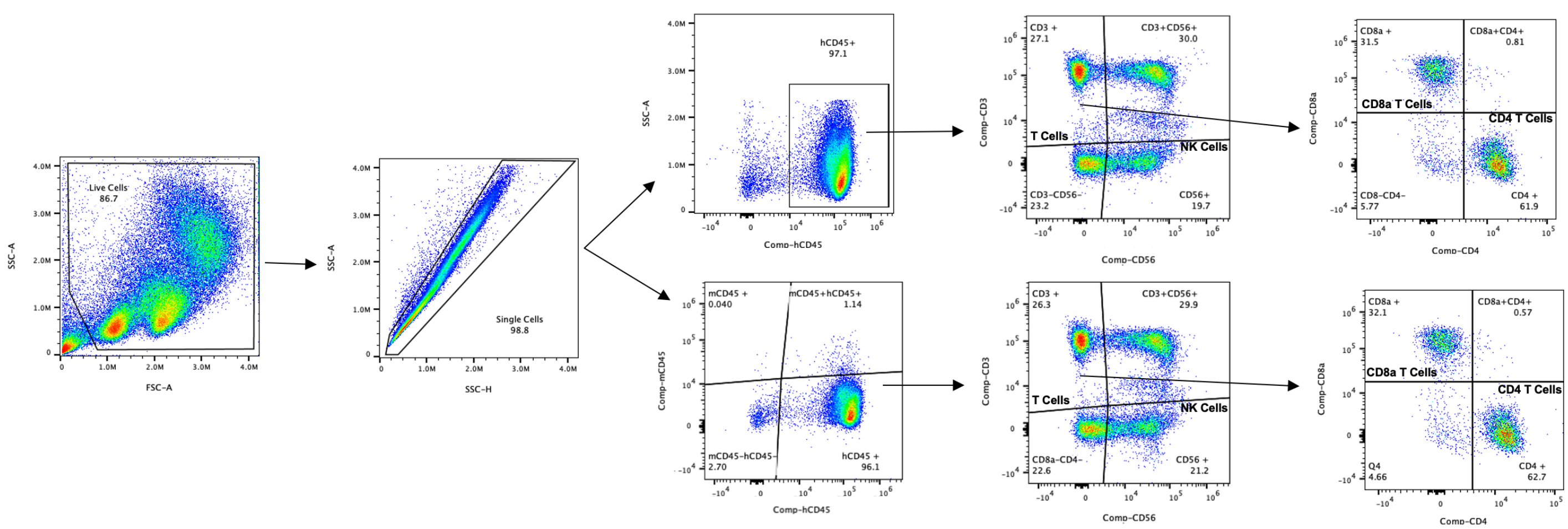

FMOs

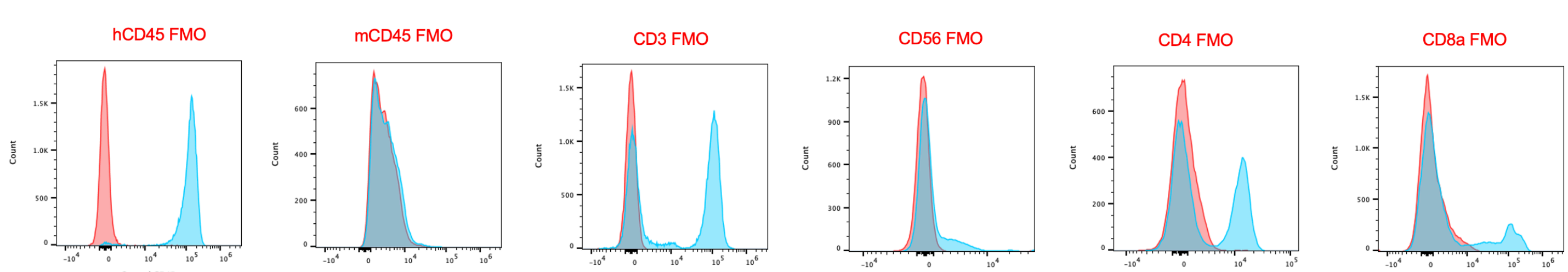

Table 2

Source and Metadata for Figure 1

SRX_accession	Tissue	Disease	Disease (Specific)	Treatment	Perturbation	Sex (Determined)
SRX23430278	Peripheral Blood Mononuclear Cells (PBMC)	HIV	HIV infection	Unsure	TotalSeq-B human hashtag antibodies and CITE-seq antibodies	Y-Linked (Male)
SRX23430277	PBMCs	HIV-B	HIV-B	Unsure	Unsure	Y-Linked (Male)
SRX18840319	Frontal cortex (BA9)	HIV	HIV infection (latent reservoir in the brain)	Antiretroviral (ART)	Antiretroviral therapy	Y-Linked (Male)
SRX21484394	CD4+ T cells	HIV	HIV	Electro-shock	Low-intensity pulse-based transcranial electrical stimulation	Unsure
SRX19758742	PBMC	HIV-1	HIV-1 infection	Antiretroviral (ART) and TLR9	Antiretroviral and TLR9 agonist treatment	Unsure
SRX18840318	Frontal cortex (BA9)	HIV	HIV persistence in the brain	Antiretroviral (ART)	Antiretroviral therapy	Unsure
SRX23408732	Peripheral Blood Mononuclear Cells (PBMC)	HIV-1	HIV-1 infected patients	Antiretroviral (ART)	Patients receiving antiretroviral therapy (ART)	Y-Linked (Male)
SRX23430279	PBMCs	HIV	HIV	Unsure	Unsure	Y-Linked (Male)
SRX20539732	Brain	HIV-associated neurocognitive dysfunction (HAND)	HIV-associated neurocognitive dysfunction (HAND)	CD4 Activation	CD3+ cell depletion, CD11b positive selection	X-Linked (Female)
SRX21215737	PBMCs	HIV-1	HIV-1 infection	Resveratrol and Pterostilbene	Resveratrol and Pterostilbene treatment	Y-Linked (Male)
SRX20542621	Peripheral Blood Mononuclear Cells (PBMC)	HIV-1	Acute HIV-1 infection	Unsure	Unsure	Y-Linked (Male)
SRX20542623	Peripheral Blood Mononuclear Cells (PBMC)	HIV-1	Acute HIV-1 infection	Untreated	None	Y-Linked (Male)
SRX21414456	Peripheral blood mononuclear cells	HIV/HBV co-infection	HIV/HBV co-infection	Antiretroviral (ART)	Antiviral therapy	Unsure
SRX21870158	Peripheral Blood Mononuclear Cells (PBMC)	HIV	Chronic HIV	Antiretroviral (ART)	Stable on ART with suppressed viral loads	Y-Linked (Male)

SRX21414450	Peripheral blood mononuclear cells	HIV/HBV co-infection	HIV/HBV co-infection	Antiretroviral (ART)	Antiviral therapy	Unsure
SRX21870170	Peripheral Blood Mononuclear Cells (PBMC)	HIV	Chronic HIV	Unsure	Unsure	Y-Linked (Male)
SRX21414454	Peripheral blood mononuclear cells	HIV/HBV co-infection	HIV/HBV co-infection	Antiretroviral (ART)	Antiviral therapy	Unsure
SRX21870147	PBMC	HIV	Chronic HIV	Unsure	Unsure	Y-Linked (Male)
SRX21870155	Peripheral Blood Mononuclear Cells (PBMC)	HIV	Chronic HIV	Unsure	Unsure	Y-Linked (Male)
SRX20542616	Peripheral blood mononuclear cells	HIV-1	acute HIV-1 infection	Untreated	peer-led intervention to increase physical activity	Unsure
SRX21870162	Peripheral Blood Mononuclear Cells (PBMC)	HIV	Chronic HIV	Antiretroviral (ART)	Antiretroviral Therapy (ART)	Unsure
SRX21870115	Peripheral Blood Mononuclear Cells (PBMC)	HIV	Chronic HIV	Unsure	Unsure	Unsure
SRX21870166	Blood	HIV	HIV, asymptomatic, healthy controls	Antiretroviral (ART)	ART treatment, longitudinal study	Y-Linked (Male)
SRX21870172	Cerebrospinal fluid	HIV	HIV infection	Antiretroviral (ART)	Antiretroviral therapy	Y-Linked (Male)
SRX21870174	Cerebrospinal fluid	HIV	HIV infection	Antiretroviral (ART)	Antiretroviral therapy	Unsure
SRX21870179	Peripheral Blood Mononuclear Cells (PBMC)	HIV	Chronic HIV	Unsure	Unsure	Unsure
SRX21870183	Cerebrospinal fluid	HIV	chronic HIV	Unsure	Unsure	Y-Linked (Male)
SRX21870157	CSF cells	HIV	Chronic HIV	Antiretroviral (ART)	Asymptomatic people living with HIV on ART	Unsure
SRX21870153	Peripheral Blood Mononuclear Cells (PBMC)	Chronic HIV, Breast cancer	Chronic HIV, Breast cancer	Unsure	Unsure	Y-Linked (Male)
SRX23454813	Blood	HIV	HIV Exposed Infected (HEI)	Unsure	Unsure	Unsure

SRX21870121	PBMC	HIV	Chronic HIV	Unsure	Unsure	Y-Linked (Male)
SRX21870122	PBMC	HIV	chronic HIV	Unsure	Unsure	Unsure
SRX21870185	Peripheral Blood Mononuclear Cells (PBMC)	HIV	Chronic HIV	Unsure	Unsure	Y-Linked (Male)
SRX17100999	Brain	HIV-1	HIV-1 infection	Efavirenz (EFV) and Nevirapine (NVP)	Efavirenz (EFV) and Nevirapine (NVP) treatments	Unsure
SRX23430276	Peripheral Blood Mononuclear Cells (PBMCs)	HIV-A	HIV-A	Unsure	No explicit treatment or perturbation beyond the context provided; although the abstract mentions suppression of HIV replication by antiretroviral therapy (ART) and use of sample barcoding for immune cell subtyping, no distinct treatment field was annotated.	Unsure
SRX19758743	PBMC (Peripheral Blood Mononuclear Cells)	HIV-1	HIV-1 infection	Antiretroviral (ART) and TLR9	Antiretroviral treatment, TLR9 agonist treatment	Unsure
SRX19838230	Blood	HIV-related comorbidities	HIV-related comorbidities, chronic inflammation	CBD	Cannabidiol (CBD) treatment	X-Linked (Female)
SRX20542624	Peripheral Blood Mononuclear Cells (PBMC)	HIV-1	Acute HIV-1 infection	Unsure	Acute HIV-1 infection context	Y-Linked (Male)
SRX21870171	PBMC	HIV	chronic HIV	Unsure	Unsure	Unsure
SRX23454805	Blood	HIV	HIV Exposed Infected (HEI)	Unsure	Unsure	Unsure
SRX18661444	Blood	HIV-1	HIV-1 infection	Antiretroviral (ART)	ART (Antiretroviral Therapy)	Unsure
SRX20642662	CD4 T cells	HIV-1	HIV-1 infection in the context of opioid addiction treatment	Opioid replacement	Opioid replacement therapy	Unsure

SRX21414444	Peripheral blood mononuclear cells	HIV/HBV co-infection	HIV/HBV co-infection	Antiretroviral (ART)	Antiviral therapy	Y-Linked (Male)
SRX21870145	PBMC (Peripheral Blood Mononuclear Cells)	HIV	Chronic HIV	Unsure	Unsure	Unsure
SRX21870123	Peripheral Blood Mononuclear Cells (PBMC)	HIV	Chronic HIV	Unsure	Unsure	Unsure
SRX20542628	PBMC	HIV-1	Acute HIV-1 infection	Unsure	Unsure	Unsure
SRX21870143	CSF	HIV	Chronic HIV	Antiretroviral (ART)	ART (Antiretroviral Therapy)	Unsure
SRX20542632	Peripheral Blood Mononuclear Cells (PBMC)	HIV-1	Acute HIV-1 infection	Unsure	Unsure	Unsure
SRX21870163	Blood	HIV	HIV infection	Untreated	Pre-ART condition	Unsure
SRX20642665	CD4 cells	HIV-1	HIV-1 infection	Opioid replacement	Opioid replacement therapy	Unsure
SRX21762248	Primary CD4 cells	HIV	HIV latency	Untreated	DMSO (control), treated with HIV latency reversal agents (SAHA/VOR, Prostratin, iBET151)	X-Linked (Female)
SRX19838229	Blood	HIV	HIV	Untreated	baseline (before CBD treatment)	X-Linked (Female)
SRX23408731	Peripheral Blood Mononuclear Cells (PBMC)	HIV-1	HIV-1 infected patients	Antiretroviral (ART)	ART (Antiretroviral Therapy)	Unsure
SRX19838231	Blood	HIV	HIV	CBD	CBD treatment	Y-Linked (Male)
SRX21870160	Cerebrospinal fluid (CSF)	HIV	Chronic HIV	Antiretroviral (ART)	Stable on ART with suppressed plasma viral loads	Y-Linked (Male)
SRX21870186	Peripheral blood mononuclear cells (PBMCs)	HIV	HIV infection	Antiretroviral (ART)	Antiretroviral therapy (ART)	Unsure
SRX21870114	Peripheral blood / PBMC	HIV	HIV infection (chronic HIV)	Antiretroviral (ART)	Antiretroviral therapy (ART)	Unsure
SRX20542630	Peripheral Blood Mononuclear Cells (PBMC)	HIV-1	Acute HIV-1 infection	Unsure	Unsure	Y-Linked (Male)

SRX21870168	Cerebrospinal fluid	HIV	chronic HIV	Unsure	Unsure	Y-Linked (Male)
SRX21870149	Peripheral blood	HIV	HIV	Antiretroviral (ART)	Antiretroviral therapy	Unsure
SRX21870111	Cerebrospinal fluid and blood	HIV	asymptomatic people living with HIV	Antiretroviral (ART)	stable on ART, One newly diagnosed not on ART	Unsure
SRX21870112	Cerebrospinal fluid (CSF)	HIV	HIV infection	Antiretroviral (ART)	Antiretroviral therapy (ART)	Unsure
SRX20642667	CD4+ T cells from peripheral blood	HIV-1	HIV-1 infection, opioid use/addiction	Opioid replacement	Opioid replacement therapy (methadone, buprenorphine with naloxone, naltrexone)	Y-Linked (Male)
SRX21870177	Cerebrospinal fluid	HIV	chronic HIV	Unsure	Unsure	Y-Linked (Male)
SRX21870116	PBMC	HIV	Chronic HIV	Antiretroviral (ART)	Participants were stable on ART with suppressed plasma viral loads.	Y-Linked (Male)
SRX14968954	Peripheral Blood Mononuclear Cells (PBMC)	HIV-1	HIV-1 infection	Unsure	Cells isolated from HIV-1-infected patients during primo-infection	Unsure
SRX23454815	Blood-derived cells	HIV	HIV Exposed Infected (HEI)	Unsure	Unsure	Unsure
SRX21870148	Peripheral Blood Mononuclear Cells (PBMC), CD4 enriched	HIV	Chronic HIV	Unsure	Unsure	Y-Linked (Male)
SRX21870176	PBMC	HIV	chronic HIV	Unsure	Unsure	Unsure
SRX21870117	Peripheral blood	HIV	HIV	Antiretroviral (ART)	Antiretroviral therapy	Y-Linked (Male)
SRX14840563	meninges	HIV-1	HIV-1 infection	Unsure	Recombinant vesicular stomatitis virus (rVSV)	Unsure
SRX21870159	Peripheral blood mononuclear cells (PBMCs)	HIV	HIV infection	Untreated	Newly Diagnosed with HIV, not yet on ART at baseline	Unsure
SRX21484382	Peripheral blood	HIV	HIV latency	CD4 Activation	CD4+ T cells activated using anti-CD3/anti-CD28 antibodies	Unsure
SRX21484393	Peripheral blood	HIV	HIV	CD4 Activation	CD4+ T cells activated using anti-CD3/anti-CD28 antibodies	Y-Linked (Male)

SRX21870181	Peripheral blood	HIV	HIV infection	Antiretroviral (ART)	Antiretroviral therapy (ART)	Unsure
SRX20326044	Tonsils	HIV-1	HIV-1 exposed	Unsure	HIV-1 infection in human tonsil explants	Unsure
SRX21870167	Blood	HIV	HIV	Antiretroviral (ART)	Antiretroviral treatment	Unsure
SRX20673992	CD4+ T cell	HIV-1	Acute HIV-1 infection	Untreated	None	Unsure
SRX21870189	Peripheral blood mononuclear cells (PBMCs)	HIV	Chronic HIV / people living with HIV (PWH)	Antiretroviral (ART)	Participants were on antiretroviral therapy (ART)	Unsure
SRX20672213	CD4-CD8- T cell	HIV-1	Acute HIV-1 infection	Unsure	Unsure	Unsure
SRX20673994	CD4+ T cell	HIV-1	Acute HIV-1 infection	Untreated	None	Unsure
SRX21870125	Blood, cerebrospinal fluid	HIV	HIV	Antiretroviral (ART)	Antiretroviral therapy	Unsure
SRX21870119	CSF	HIV	Chronic HIV	Antiretroviral (ART)	Stable on ART, one newly diagnosed not on ART	Unsure
SRX21870188	Peripheral Blood Mononuclear Cells (PBMC)	HIV	Chronic HIV	Unsure	Unsure	Y-Linked (Male)
SRX21762249	Primary CD4 cells	HIV	HIV latency	iBET151	iBET151	X-Linked (Female)
SRX20326043	Tonsils	HIV-1	HIV-1 infection (unexposed)	Unsure	HIV-1 infection (ex vivo in tonsil explants)	Unsure
SRX21484392	Peripheral blood	HIV	HIV	IL-10	Gene transfer of interleukin-10 (IL-10)	Y-Linked (Male)
SRX21870154	Peripheral blood	HIV	HIV infection	Antiretroviral (ART)	Antiretroviral therapy (ART) status	Unsure
SRX20326046	Tonsils	HIV-1	HIV-1 exposure	Unsure	Exposed to HIV-1 in tonsil explants	X-Linked (Female)
SRX19758737	PBMC (Peripheral Blood Mononuclear Cells)	HIV-1	HIV-1 infection	Antiretroviral (ART) and TLR9	Antiretroviral and TLR9 agonist treatment	Y-Linked (Male)
SRX21870146	PBMC (blood)	HIV	Chronic HIV	Unsure	Unsure	Unsure
SRX15446293	PBMC	HIV-1	HIV-1 infected, aviraemic, on ART	Antiretroviral (ART)	Antiretroviral therapy (ART)	Y-Linked (Male)

SRX21870120	Cerebrospinal fluid	HIV	Chronic HIV	Unsure	Fresh CSF and blood processed within one hour of collection; CSF centrifugation at 300g for 10 minutes; sample prepared for single-cell RNA/TCR sequencing using the 10X Genomics system (includes subjects on antiretroviral therapy)	Unsure
SRX21870184	Cerebrospinal fluid (CSF)	HIV	HIV infection	Antiretroviral (ART)	Antiretroviral therapy (ART)	Unsure
SRX21870152	CSF	HIV	Chronic HIV	Unsure	Unsure	Unsure
SRX23454804	Peripheral Blood Mononuclear Cells (PBMC)	HIV	HIV-related context	Antiretroviral (ART)	Early antiretroviral therapy (ART) initiation	Unsure
SRX21870165	Cerebrospinal fluid	HIV	HIV	Antiretroviral (ART)	Antiretroviral therapy	Unsure
SRX20673995	CD4+ T cell	HIV-1	Acute HIV-1 infection	Unsure	Unsure	Unsure
SRX21484385	Peripheral blood	HIV	HIV latency	CD4 Activation	CD4+ T cells activated using anti-CD3/anti-CD28 antibodies; direct infection with CXCR4- and CCR5...	X-Linked (Female)
SRX14969037	PBMC	HIV-1	HIV-1 exposure	Antiretroviral (ART	HIV-1 NL-AD8 with Vpx, AZT/NVP	Y-Linked (Male)
SRX20673991	CD4+ T cell	HIV-1	Acute HIV-1 infection	Unsure	Unsure	Unsure
SRX20672219	CD4+ T cell	HIV-1	Acute HIV-1 infection	Unsure	Unsure	Y-Linked (Male)
SRX20326040	Tonsils	HIV-1	HIV-1 infection (exposed)	Unsure	HIV-1 exposure	Y-Linked (Male)
SRX23454816	Blood	HIV	HIV Exposed Infected (HEI)	Untreated	Not available	Unsure
SRX23454811	PBMC (peripheral blood mononuclear cells)	HIV	HIV infection	Untreated	None	Unsure

SRX23454812	PBMC (peripheral blood mononuclear cells)	HIV	HIV Exposed Infected (HEI)	Unsure	Unsure	Y-Linked (Male)
SRX21870180	Peripheral Blood Mononuclear Cells (PBMC)	HIV	Chronic HIV	Antiretroviral (ART)	Stable on ART with suppressed plasma viral loads	Y-Linked (Male)
SRX20326042	Tonsils	HIV-1	exposed to HIV-1	Unsure	HIV-1 infection in tonsil explants	Unsure
SRX21870110	PBMC	HIV	chronic HIV	Unsure	Unsure	Y-Linked (Male)
SRX15446295	PBMC	HIV-1	HIV-1 infection	Antiretroviral (ART)	Antiretroviral therapy (ART)	Y-Linked (Male)
SRX21870178	Cerebrospinal fluid	HIV	HIV infection	Antiretroviral (ART)	Antiretroviral therapy	Unsure
SRX21870182	PBMC	HIV	chronic HIV	Unsure	Unsure	Unsure
SRX21870161	Cerebrospinal fluid (CSF) cells	HIV	HIV	Antiretroviral (ART)	Antiretroviral therapy (ART)	Unsure
SRX19758740	PBMC	HIV-1	HIV-1 infection	Antiretroviral (ART) and TLR9	Antiretroviral treatment, TLR9 agonist treatment	Unsure
SRX21870156	Blood	HIV	HIV infection	Unsure	Unsure	Unsure
SRX15446294	PBMC	HIV-1	HIV-1 infected, aviraemic, ART	Unsure	Cells isolated by magnetic-activated cell sorting and treated with various antibodies	Unsure
SRX20672214	CD4+ T cell	HIV-1	Acute HIV-1 infection	Untreated	None	Y-Linked (Male)
SRX21870151	CSF (Cerebrospinal Fluid)	HIV	Chronic HIV	Antiretroviral (ART)	Not specified; participants were stable on ART (Antiretroviral Therapy)	Y-Linked (Male)
SRX20672216	CD4+ T cell	HIV-1	Acute HIV-1 infection	Untreated	None	Y-Linked (Male)
SRX20673993	CD4+ T cell	HIV-1	Acute HIV-1 infection	Unsure	Unsure	Y-Linked (Male)
SRX19970323	Peripheral blood	HIV-TB co-infection	HIV-TB co-infection	Unsure	hematopoietic Stem cell transplant	unsure
SRX18295262	blood	HIV/AIDS and malaria	HIV/AIDS and malaria	CYP2B6-metabolized drugs	CYP2B6-metabolized drugs	Y-Linked (Male)
SRX19758748	Peripheral blood mononuclear cells	HIV-1	HIV-1 infection	Antiretroviral (ART) and TLR9	Antiretroviral treatment, TLR9 agonist treatment	Unsure
SRX25507069	Bronchoalveolar lavage	HIV-TB co-infection	Mycobacterium tuberculosis infection in people living with HIV	Antiretroviral (ART)	Mtb strain H37Rv at MOI 6.5:1, ART TDF_FTC_EFV	X-Linked (Female)

SRX24475787	Bronchoalveolar lavage (BAL) cells	HIV-TB co-infection	HIV, tuberculosis	Antiretroviral (ART)	Anti-retroviral therapy, Mycobacterium tuberculosis infection	Unsure
SRX21870187	Peripheral blood mononuclear cells	HIV	chronic HIV	Antiretroviral (ART)	Stable on ART with suppressed viral loads	Y-Linked (Male)
SRX20542619	Peripheral Blood Mononuclear Cells (PBMC)	HIV-1	Acute HIV-1 infection	Unsure	Unsure	Unsure
SRX9106611	Peripheral blood mononuclear cells (PBMCs)	HIV	HIV infection and healthy controls	Untreated	None	Y-Linked (Male)
SRX10235924	Peripheral blood mononuclear cells (PBMCs)	HIV-1	none (healthy, HIV-1 negative)	Unsure	None reported	Unsure
SRX21414452	Peripheral blood mononuclear cells	HIV/HBV co-infection	HIV/HBV co-infection	Unsure	Unsure	Y-Linked (Male)
SRX20542633	Peripheral Blood Mononuclear Cells (PBMC)	HIV-1	Acute HIV-1 infection	Unsure	Unsure	Unsure
SRX20542637	PBMC (Peripheral Blood Mononuclear Cells)	HIV-1	Acute HIV-1 infection	Untreated	None	Unsure
SRX20542635	Peripheral Blood Mononuclear Cells (PBMC)	HIV-1	Acute HIV-1 infection	Unsure	Acute HIV-1 infection	Y-Linked (Male)
SRX21870113	Peripheral Blood Mononuclear Cells (PBMC)	HIV	Chronic HIV	Antiretroviral (ART)	Stable on ART, suppressed viral loads	Y-Linked (Male)
SRX20461176	Peripheral blood mononuclear cells	HIV	HIV infections	Unsure	Stimulated PBMCs	Unsure
SRX20542618	Peripheral Blood Mononuclear Cells (PBMC)	HIV-1	Acute HIV-1 infection	Unsure	Unsure	Unsure
SRX21870164	Cerebrospinal fluid	HIV	chronic HIV	Antiretroviral (ART)	Stable on ART	Y-Linked (Male)

SRX24098260	Peripheral blood mononuclear cells (PBMC)	Chronic visceral leishmaniasis with HIV co-infection	chronic visceral leishmaniasis, HIV coinfection	Unsure	Unsure	Y-Linked (Male)
SRX21870175	Peripheral Blood Mononuclear Cells (PBMC)	HIV	Chronic HIV	Antiretroviral (ART)	Asymptomatic People With HIV (PWH) on ART	Unsure
SRX9632954	PBMC	HIV-1	HIV-1 infection	Antiretroviral (ART)	HIV-1 ART treated	X-Linked (Female)
SRX20542614	Peripheral Blood Mononuclear Cells (PBMC)	HIV-1	Acute HIV-1 infection	Unsure	Unsure	Unsure
SRX9632946	Peripheral Blood Mononuclear Cells (PBMC)	HIV-1	HIV-1 ART treated	Antiretroviral (ART)	HIV-1 ART (Antiretroviral Therapy)	Y-Linked (Male)
SRX9106608	Peripheral blood mononuclear cells (PBMCs)	HIV	HIV infection and healthy controls	Untreated	None	Unsure
SRX9632947	PBMC (Peripheral Blood Mononuclear Cells)	HIV-1	HIV-1 untreated	Untreated	Untreated	Y-Linked (Male)
SRX24098284	Peripheral blood mononuclear cells (PBMC)	Chronic visceral leishmaniasis with HIV co-infection	Chronic visceral leishmaniasis with HIV co-infection	Unsure	None specified	Unsure
SRX21414448	Peripheral blood mononuclear cells	HIV/HBV co-infection	HIV/HBV co-infection	Unsure	Chronic stress	Y-Linked (Male)
SRX9632956	Peripheral Blood Mononuclear Cells (PBMC)	HIV-1	HIV-1 seronegative	Unsure	Unsure	Y-Linked (Male)
SRX20542617	Peripheral Blood Mononuclear Cells (PBMC)	HIV-1	Acute HIV-1 infection	Warfarin	Anticoagulants (specifically Warfarin)	Y-Linked (Male)
SRX21414440	Peripheral blood mononuclear cells	HIV/HBV co-infection	HIV/HBV co-infection	Antiretroviral (ART)	Antiviral therapy	Unsure
SRX9632951	PBMC (Peripheral Blood Mononuclear Cells)	HIV-1	HIV-1 seronegative	Unsure	Unsure	Unsure

SRX24475798	Pronchoalveolar lavage	HIV	People Living with HIV (PLWH)	Mtb H37Rv	Post-infection with Mtb H37Rv	Y-Linked (Male)
SRX21051459	Peripheral Blood Mononuclear Cells (PBMC)	HIV	HIV	Resiquimod ATL-DC	Resiquimod, ATL-DC vaccination	Y-Linked (Male)
SRX19838232	Blood	HIV	HIV	CBD	CBD (Cannabidiol)	Y-Linked (Male)
SRX16745147	Nuclei isolated from 2D10 cells	HIV	Latent HIV infection	SAHA	SAHA treatment	Y-Linked (Male)
SRX20051653	Blood PBMCs	HIV	HIV and glucose intolerance	Unsure	Unsure	Y-Linked (Male)
SRX20542622	Peripheral Blood Mononuclear Cells (PBMC)	HIV-1	Acute HIV-1 infection	Untreated	Control: 11 mM glucose; Treatment: 25 mM glucose for 1 day and 2 days	Y-Linked (Male)
SRX21414446	Peripheral blood mononuclear cells	HIV/HBV co-infection	HIV/HBV co-infection	Antiretroviral (ART)	Antiviral therapy	Unsure
SRX20542627	Peripheral Blood Mononuclear Cells (PBMC)	HIV-1	Acute HIV-1 infection	Untreated	None	Y-Linked (Male)
SRX20542634	PBMC (Peripheral Blood Mononuclear Cells)	HIV-1	Acute HIV-1 infection	Untreated	None	Unsure
SRX9106610	Peripheral blood mononuclear cells (PBMCs)	HIV	HIV infection	Unsure	None specified	Unsure
SRX20542636	Peripheral Blood Mononuclear Cells (PBMC)	HIV-1	Acute HIV-1 infection	Unsure	Unsure	Y-Linked (Male)
SRX20542620	Peripheral blood mononuclear cells	HIV-1	acute HIV-1 infection	Unsure	Unsure	Unsure
SRX25507062	Bronchoalveolar lavage	HIV-TB co-infection	latent tuberculosis infection, HIV	Mtb H37Rv	Mtb strain H37Rv challenge	Unsure
SRX20733028	Breast	HIV	HIV-associated cognitive impairment	Antiretroviral (ART)	Antiretroviral therapy (HAART)	Unsure
SRX20542631	Peripheral Blood Mononuclear Cells (PBMC)	HIV-1	Acute HIV-1 infection	Unsure	Unsure	Y-Linked (Male)

SRX20542615	Peripheral Blood Mononuclear Cells (PBMC)	HIV-1	Acute HIV-1 infection	Unsure	Unsure	Unsure
SRX24098271	Peripheral blood mononuclear cells (PBMC)	Chronic visceral leishmaniasis with HIV co-infection	chronic visceral leishmaniasis, HIV coinfection	Unsure	None reported	Unsure
SRX9106609	Peripheral blood mononuclear cells (PBMCs)	HIV	HIV infection (plus healthy controls)	Untreated	None	Unsure
SRX20542625	Peripheral Blood Mononuclear Cells (PBMC)	HIV-1	Acute HIV-1 infection	Untreated	None	Y-Linked (Male)
SRX21414458	Peripheral blood mononuclear cells	HIV/HBV co-infection	HIV/HBV co-infection	Antiretroviral (ART)	Antiviral therapy	Unsure
SRX8680765	Peripheral blood monocytes	HIV	HIV infection	Antiretroviral (ART)	ART treatment, HIV infection	X-Linked (Female)
SRX9632950	PBMC	HIV-1	HIV-1 infection	Antiretroviral (ART)	HIV-1 ART treated	Unsure
SRX24098265	Peripheral blood mononuclear cells (PBMC)	Chronic visceral leishmaniasis with HIV co-infection	chronic visceral leishmaniasis, HIV coinfection	Unsure	Unsure	Y-Linked (Male)
SRX20051654	Blood PBMCs	HIV	HIV and glucose intolerance	Unsure	Unsure	Unsure
SRX24098262	Peripheral blood mononuclear cells (PBMC)	Chronic visceral leishmaniasis with HIV co-infection	chronic visceral leishmaniasis and HIV co-infection	Unsure	None reported	Unsure
SRX9632948	PBMC	HIV-1	HIV-1 untreated	Untreated	Not applicable	Unsure
SRX14036492	Jurkat T-cell leukemia cell line (N6 clone)	HIV	HIV latency model (no primary patient samples)	iBET151	500 nM iBET151 + 100 nM AZD5582	Unsure
SRX25507053	Bronchoalveolar lavage	HIV	HIV positive, long-term ART, no TB history	MtbINF	MtbINF at MOI 6.5:1, 6 hours incubation	Unsure
SRX19838227	Blood	HIV-related comorbidities	HIV-related comorbidities	CBD	Baseline (no treatment), CBD treatment (after at least one month)	Unsure
SRX27129497	Blood	HIV	HIV infections, depression, anxiety, PTSD	Unsure	Initiating HIV care in Cameroon	Unsure
SRX14036491	Not_applicable (Jurkat T cell line)	HIV	HIV latency model	iBET151	iBET151 (500 nM)	Unsure

SRX25335002	Peripheral blood mononuclear cells	HIV Type-2 Diabetes	Type 2 Diabetes, HIV Infections	Anti-PD-1 and Anti-CTLA-4	Anti-PD-1 and Anti-CTLA-4 therapies	Unsure
SRX9106607	Peripheral blood mononuclear cells (PBMCs)	HIV	HIV infection (plus healthy control)	KLRG1 Blockade	Ex-vivo antibody blockade of KLRG1	Unsure
SRX9632952	PBMC	HIV-1	HIV-1 untreated	Unsure	HIV-1 infection	Unsure
SRX9106606	Peripheral blood mononuclear cells (PBMCs)	HIV	HIV infection	Unsure	None reported	Unsure
SRX9632945	PBMC	HIV-1	HIV-1 untreated	Untreated	Not applicable	Unsure
SRX21484390	Peripheral blood	HIV	HIV latency	CD4 Activation	CD4+ T cells activated with anti-CD3/anti-CD28 antibodies	Unsure
SRX25507049	Bronchoalveolar lavage	HIV-TB co-infection	Mycobacterium tuberculosis infection in people with HIV	Mtb H37Rv	Media + Mtb strain H37Rv	X-Linked (Female)
SRX27383476	Cervical spinal cord	HIV-associated neurocognitive dysfunction (HAND)	HIV/AIDS with neurological diseases	CHM	Chinese herbal medicine (CHM)	X-Linked (Female)
SRX25507043	Bronchoalveolar lavage	HIV-TB co-infection	latent TB infection, HIV	Mtb H37Rv	Mtb strain H37Rv challenge	Unsure
SRX19758738	Peripheral Blood Mononuclear Cells (PBMC)	HIV-1	HIV-1 infection	Antiretroviral (ART) and TLR9	Antiretroviral and TLR9 agonist treatment	X-Linked (Female)
SRX20542629	Peripheral Blood Mononuclear Cells (PBMC)	HIV-1	Acute HIV-1 infection	Unsure	Unsure	Unsure
SRX24475797	Bronchoalveolar lavage	HIV	HIV	Untreated	Uninfected	Y-Linked (Male)
SRX24475788	Bronchoalveolar lavage	HIV	PLWH (People Living With HIV)	Unsure	Post-infection with Mtb (Mycobacterium tuberculosis)	Y-Linked (Male)
SRX20542626	Peripheral Blood Mononuclear Cells (PBMC)	HIV-1	Acute HIV-1 infection	Unsure	Unsure	Unsure
SRX20326047	Tonsils	HIV-1	HIV-1 exposure	Unsure	HIV-1 infection	Unsure
SRX25507032	Bronchoalveolar lavage	HIV-TB co-infection	HIV, latent TB infection	Mtb H37Rv	Mtb strain H37Rv challenge	X-Linked (Female)

SRX25507048	Bronchoalveolar lavage	HIV	HIV positive, no history of tuberculosis despite long-term exposure to Mycobacterium tuberculosis	Antiretroviral (ART)	MtbNEG (not challenged), ART: ABC_3TC_NVP, Years on ART: 12	Unsure
SRX20672220	CD4+ T cell	HIV-1	Acute HIV-1 infection	Untreated	None	Y-Linked (Male)
SRX21227449	Memory CD4+ T cells	HIV-1	HIV-1-infected	Antiretroviral (ART)	Antiretroviral therapy	Unsure
SRX24475786	Bronchoalveolar lavage	HIV	People Living with HIV (PLWH)	Mtb H37Rv	Cells infected with Mtb H37Rv at an MOI of 2:1	Y-Linked (Male)
SRX20673996	CD4+ T cell	HIV-1	Acute HIV-1 infection	Unsure	Unsure	Unsure
SRX19758746	PBMC (Peripheral Blood Mononuclear Cells)	HIV-1	HIV-1 infection	Antiretroviral (ART) and TLR9	Antiretroviral treatment, TLR9 agonist treatment	Unsure
SRX25507064	Bronchoalveolar lavage	HIV-TB co-infection	HIV, latent TB infection	Mtb	stimulated with Mycobacterium tuberculosis	X-Linked (Female)
SRX25507051	Bronchoalveolar lavage	HIV-TB co-infection	HIV, latent TB infection	Mtb H37Rv	Mtb strain H37Rv challenge, ART regimen TDF_FTC_EFV	X-Linked (Female)
SRX24098259	PBMC (peripheral blood mononuclear cells)	Chronic visceral leishmaniasis with HIV co-infection	Chronic visceral leishmaniasis with HIV coinfection	Unsure	Unsure	Unsure
SRX24098264	PBMC (peripheral blood mononuclear cells)	Chronic visceral leishmaniasis with HIV co-infection	Chronic visceral leishmaniasis, HIV coinfection	Unsure	Unsure	Unsure
SRX9632953	PBMC (Peripheral Blood Mononuclear Cells)	HIV-1	HIV-1 untreated	Untreated	Not applicable (untreated)	Unsure
SRX21484386	Peripheral blood	HIV	HIV latency	CD4 Activation	CD4+ T cells activated using anti-CD3/anti-CD28 antibodies; co-culture with autologous productive...	Unsure

SRX19758745	PBMC (Peripheral Blood Mononuclear Cells)	HIV-1	HIV-1 infection	Antiretroviral (ART) and TLR9	Antiretroviral and TLR9 agonist treatment	Y-Linked (Male)
SRX20732904	Breast	HIV	HIV and Alcohol Dependence	Unsure	Unsure	X-Linked (Female)
SRX19186264	Peripheral blood	HIV	HIV infection	CD4 Activation	AntiCD3/antiCD28 antibodies, HIV NL4-3	X-Linked (Female)
SRX25507034	Bronchoalveolar lavage	HIV-TB co-infection	HIV, latent TB infection	Mtb	ex vivo stimulated with Mycobacterium tuberculosis	X-Linked (Female)
SRX23454817	Blood	HIV	HIV Exposed Infected (HEI)	Unsure	Unsure	Unsure
SRX8680764	Peripheral blood monocytes (CD14+ CD16+)	HIV	HIV infection, HIV+ ART-treated, uninfected controls	Antiretroviral (ART)	ART treatment in subset	Unsure
SRX23454809	Blood	HIV	HIV Exposed Infected (HEI)	Unsure	Unsure	Y-Linked (Male)
SRX24475795	Bronchoalveolar lavage	HIV	PLWH (People Living With HIV)	Untreated	Uninfected	Unsure
SRX19758747	PBMC	HIV-1	HIV-1 infection	Antiretroviral (ART) and TLR9	Antiretroviral treatment, TLR9 agonist treatment	Unsure
SRX21762262	Unsure	HIV	HIV latency	iBET151	iBET151	Unsure
SRX20326038	Tonsil	HIV-1	HIV-1 infection (exposed)	Unsure	HIV-1 exposure in tonsil explants	Unsure
SRX24039024	Stem cell derived choroid plexus brain organoids	HIV	HIV infection	Untreated	mock medium (control condition)	Y-Linked (Male)
SRX9632949	PBMC	HIV-1	HIV-1 untreated	Untreated	Not applicable	Y-Linked (Male)
SRX21762251	Primary CD4 cells	HIV	HIV latency	SAHA	SAHA/VOR (HIV latency reversal agents)	Unsure
SRX24098288	Peripheral blood mononuclear cells (PBMC)	Chronic visceral leishmaniasis with HIV co-infection	chronic visceral leishmaniasis, HIV coinfection	Unsure	None specified	Y-Linked (Male)
SRX24098274	Peripheral blood mononuclear cells (PBMC)	Chronic visceral leishmaniasis with HIV co-infection	chronic visceral leishmaniasis in HIV-coinfected patients	Unsure	Unsure	Y-Linked (Male)

SRX19758739	Peripheral Blood Mononuclear Cells (PBMC)	HIV-1	HIV-1 infection	TLR9 agonist	TLR9 agonist treatment	X-Linked (Female)
SRX19186268	Peripheral blood	HIV	HIV-seronegative blood donor	CD4 Activation	antiCD3/antiCD28 antibodies, HIV NL4-3 infection	X-Linked (Female)
SRX21484380	Peripheral blood	HIV	HIV latency studies	CD4 Activation	activation of CD4+ T cells using antibodies	Unsure
SRX8680766	Peripheral blood monocytes (CD14+CD16+)	HIV	HIV infection (with uninfected controls)	Antiretroviral (ART)	Antiretroviral therapy (ART) in some HIV+ samples	Unsure
SRX24667633	Lamina Propria Aggregate Culture (LPMC)	HIV-1	HIV-1 infection	Unsure	E. coli lysate	X-Linked (Female)
SRX20672217	CD4+ T cell	HIV-1	Acute HIV-1 infection	TKI	Tyrosine kinase inhibitors (TKIs) for EGFR positive patients in lung cancer	Y-Linked (Male)
SRX21484388	Peripheral blood	HIV	HIV latency	CD4 Activation	Anti-CD3/anti-CD28 antibodies, direct infection	X-Linked (Female)
SRX14036490	Jurkat latency model and primary CD4 T cells	HIV	HIV latency/infection	iBET151	AZD5582 combined with BET inhibitors	Unsure
SRX15197395	Cerebrospinal fluid	HIV	HIV	Antiretroviral (ART)	ART (Antiretroviral Treatment)	X-Linked (Female)
SRX21762250	Primary CD4 cells	HIV	HIV latency	Prostratin	Prostratin (HIV latency reversal agent)	Unsure
SRX20673997	CD4+ T cell	HIV-1	Acute HIV-1 infection	Unsure	Unsure	Y-Linked (Male)
SRX21484395	Peripheral blood	HIV	HIV	CD4 Activation	CD4+ T cells activated using anti-CD3/anti-CD28 antibodies	Unsure
SRX21651539	Blood	HIV	HIV	Antiretroviral (ART)	Antiretroviral therapy	unsure
SRX20673998	CD4+ T cell	HIV-1	Acute HIV-1 infection	Unsure	Unsure	Y-Linked (Male)
SRX17006859	Primary CD4+ T cells	HIV	HIV latency/HIV infection	HA15	HA15 treatment and ISR/ATF4 signaling activation	X-Linked (Female)
SRX24623333	Peripheral Blood Mononuclear Cells (PBMCs)	HIV-1	HIV-1 related	CD4 Activation	Anti CD3/CD38 activation	Y-Linked (Male)

SRX24667632	Lamina Propria Aggregate Culture (LPMC)	HIV-1	related to CD4 T cells and HIV-1 infection	Unsure	E. coli lysate	X-Linked (Female)
SRX20326041	Tonsils	HIV-1	HIV-1 infection	Unsure	HIV-1 infection (ex vivo)	Y-Linked (Male)
SRX21762256	Primary CD4 cells	HIV	HIV latency	Untreated	DMSO (control), treated with HIV latency reversal agents (SAHA/VOR, prostratin, iBET151)	X-Linked (Female)
SRX21762258	Primary CD4 cells	HIV	HIV latency	SAHA	SAHA/VOR (HIV latency reversal agents)	X-Linked (Female)
SRX20672215	CD4+ T cell	HIV-1	Acute HIV-1 infection	Unsure	Unsure	Y-Linked (Male)
SRX21870124	PBMC	HIV	Chronic HIV	Antiretroviral (ART)	Stable on ART with suppressed viral loads	Unsure
SRX21484383	Peripheral blood	HIV	HIV latency	CD4 Activation	CD4+ T cells activated using anti-CD3/anti-CD28 antibodies, co-culture with autologous productive...	X-Linked (Female)
SRX24623338	CD4+ T cells	HIV-1	HIV-1 infection	Antiretroviral (ART)	Antiretroviral drugs: Tenofovir, Emtricitabine, Nevirapine, T-20	Unsure
SRX9568866	blood (primary HIV-specific memory CD8+ T cells)	HIV	HIV positive	Unsure	None specified	Unsure

Supplementary Table 3. Normalized cross-experiment CD4⁺ T-cell rescue analysis comparing PBMC-derived and HIV-Env-vaccinated humanized mouse-derived NK-cell treatments

Analysis	Blood $CD4^+$ T-cell rescue	Spleen $CD4^+$ T-cell rescue	Combined blood + spleen rescue	Interpretation
**PBMC-derived $CXCR6^+$ NK cells**	50%	49%	50%	Partial $CD4^+$ T-cell rescue
**HIV-Env–vaccinated humanized mouse liver–derived $CXCR6^+$ NK cells**	85%	122%	104%	Near-complete or complete $CD4^+$ T-cell rescue
**HIV-Env–vaccinated versus PBMC-derived $CXCR6^+$ NK cells**	$p = 0.0027$	$p = 0.00018$	$p = 2.6 \times 10^{-6}$	HIV-Env–vaccinated $CXCR6^+$ NK cells mediate significantly stronger rescue
**HIV-Env–vaccinated versus PBMC-derived $CXCR6^-$ NK cells**	$p = 0.059$	$p = 0.011$	$p = 0.0045$	NK-cell source/priming also contributes to rescue
**$CXCR6^+$-specific enhancement by NK-cell source**	$p = 0.49$	$p = 0.048$	Not reported	$CXCR6^+$-specific advantage is strongest in spleen
