# Supplementary material for: CXCR6+ natural killer cell immunotherapy preserves CD4+ T helper cells in humanized mice
Source: Front Immunol. 2026 Aug 10;17:1888755. doi: 10.3389/fimmu.2026.1888755 (PMC13501908; doi:10.3389/fimmu.2026.1888755)
Supplement: Supplementary file 2 [file DataSheet2.pdf]

Supplemental Figure 1. In vitro expansion and purity of expanded PBMC-derived CXCR6⁺ and CXCR6⁻ NK cells as determined by flow cytometry

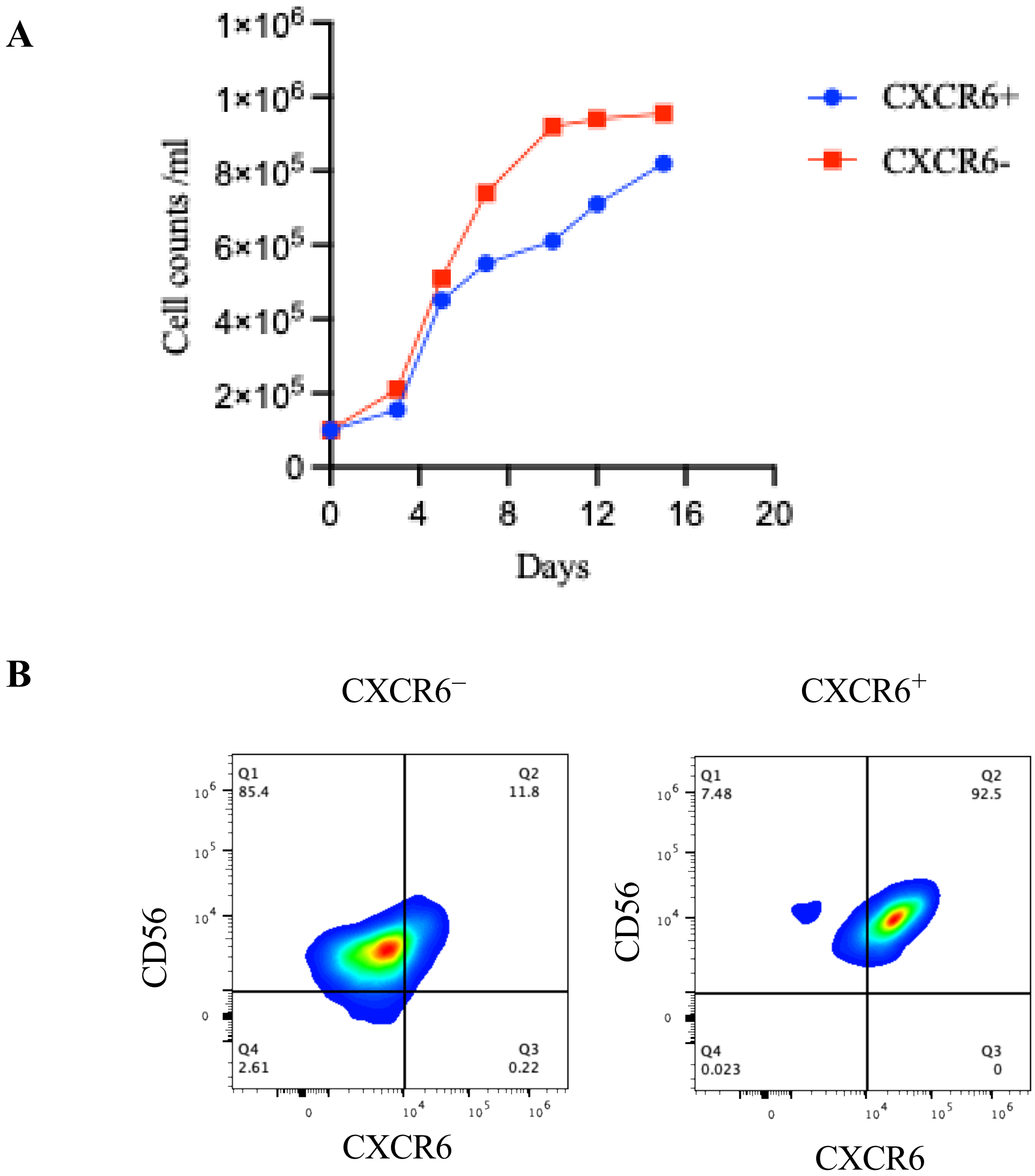

Supplemental Figure 1. In vitro expansion and post-expansion purity of PBMC-derived CXCR6⁺ and CXCR6⁻ NK cells were assessed by cell counting and flow cytometry. A) Growth kinetics of expanded CXCR6⁺ and CXCR6⁻ NK cells are shown as cell counts per milliliter over 15 days. **B)** Representative flow cytometry plots show CD56 and CXCR6 expression in expanded CXCR6⁻ and CXCR6⁺ NK cell populations as determined by flow cytometry.

Supplemental Figure 2. Experimental outline for NK cell immunotherapy using healthy donor PBMC derived NK cells

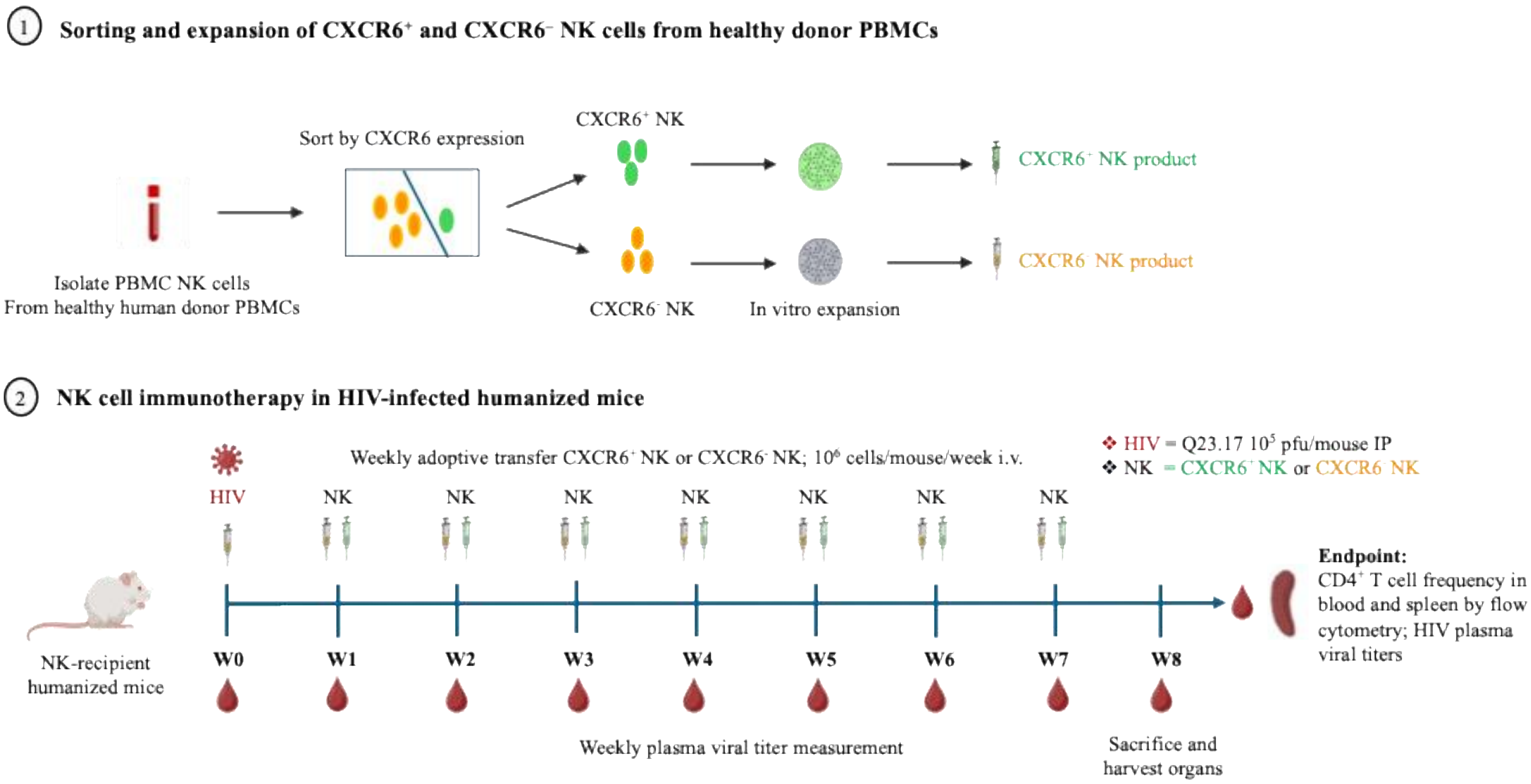

Supplemental Figure 2. Experimental outline for NK cell immunotherapy using healthy donor PBMC derived NK cells. Healthy donor PBMC derived NK cells were sorted based on CXCR6 expression and expanded in vitro to generate $CXCR6^{+}$ and $CXCR6^{-}$ NK cell products. Umbilical cord blood-derived $CD34^{+}$ HSC-humanized IL-15TgNSG mice were infected intraperitoneally with HIV-1 Q23.17, followed by weekly intravenous adoptive transfer of PBS control, 10^{6} $CXCR6^{+}$ NK cells, or 10^{6} $CXCR6^{-}$ NK cells per mouse. Plasma was collected longitudinally each week to measure HIV viral burden, and mice were sacrificed at week 8 for endpoint analysis of $CD4^{+}$ T cell preservation in blood and spleen by flow cytometry, together with assessment of plasma viral titers.

Supplemental Figure 3. In vitro expansion and post-expansion purity of HIV-Env-vaccinated humanized mouse liver-derived CXCR6⁺ and CXCR6⁻ NK cells were assessed by cell counting and flow cytometry.

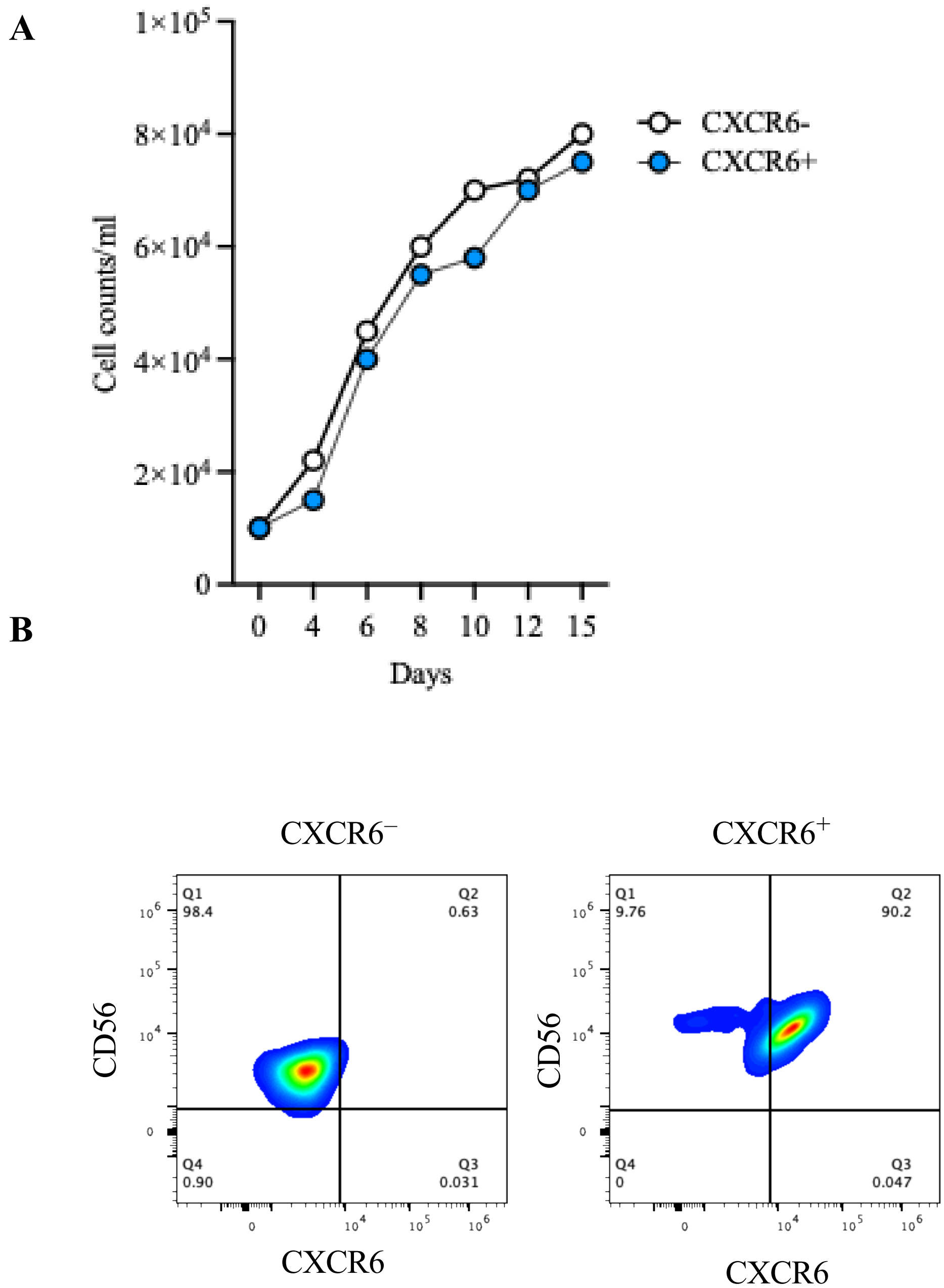

Supplemental Figure 3. In vitro expansion and post-expansion purity of HIV-Env-vaccinated humanized mouse liver-derived CXCR6⁺ and CXCR6⁻ NK cells were assessed by cell counting and flow cytometry. A) Growth kinetics of expanded CXCR6⁺ and CXCR6⁻ NK cells are shown as cell counts per milliliter over 15 days. **B)** Representative flow cytometry plots show CD56 and CXCR6 expression in expanded CXCR6⁻ and CXCR6⁺ NK cell populations as determined by flow cytometry.

Supplemental Figure 4. Experimental outline for NK cell immunotherapy using HIV Env-primed liver NK cells

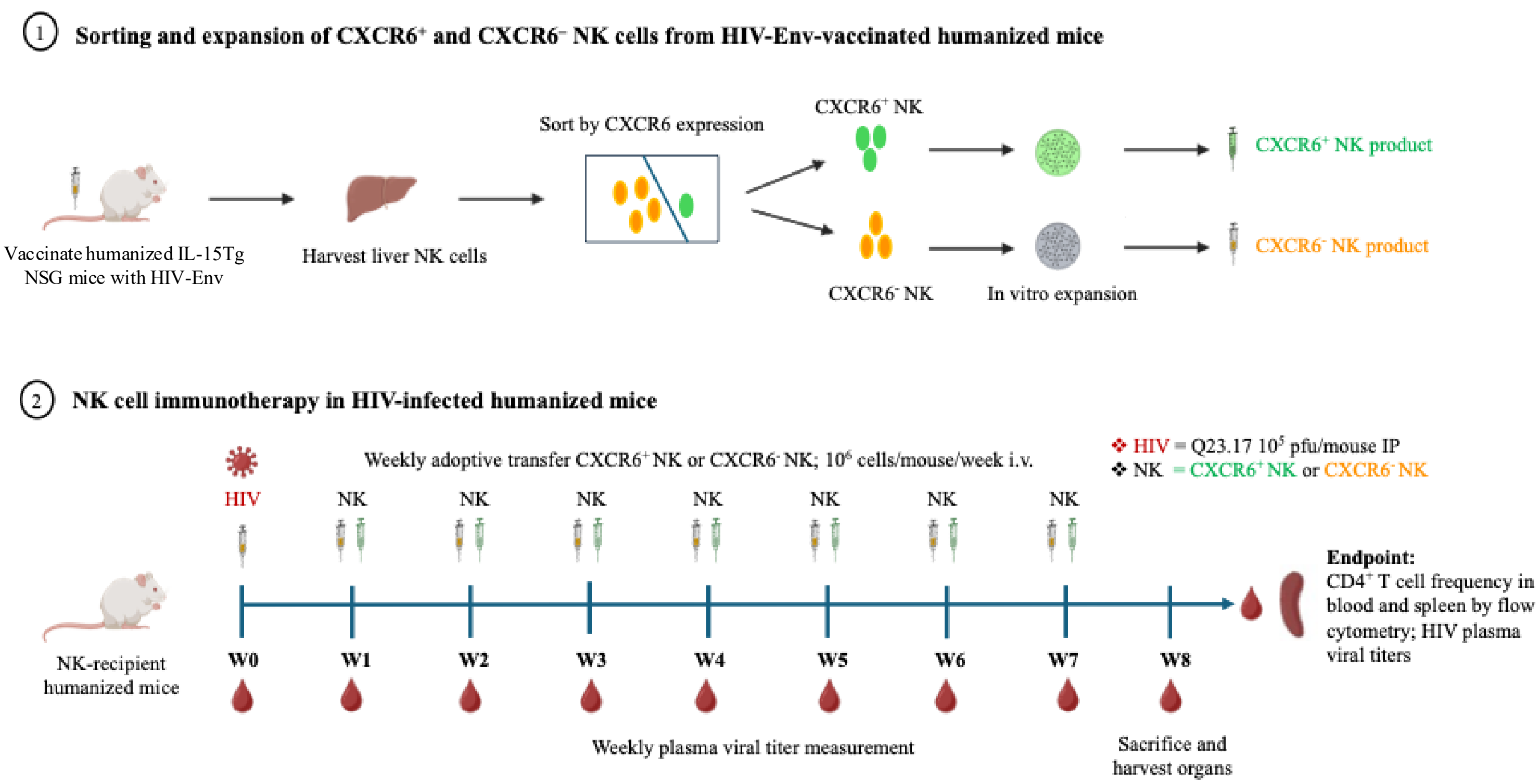

Supplemental Figure 4. Experimental outline for NK cell immunotherapy using HIV Env-primed liver NK cells. IL-15TgNSG mice were vaccinated with HIV Env, and liver NK cells were harvested, sorted based on CXCR6 expression, and expanded in vitro to generate $CXCR6^+$ and $CXCR6^-$ NK cell products. Umbilical cord blood-derived $CD34^+$ HSC-humanized IL-15TgNSG mice were infected intraperitoneally with HIV-1 Q23.17, followed by weekly intravenous adoptive transfer of PBS control, 10^6 $CXCR6^+$ NK cells, or 10^6 $CXCR6^-$ NK cells per mouse. Plasma was collected longitudinally each week to measure HIV viral burden, and mice were sacrificed at week 8 for endpoint analysis of $CD4^+$ T cell preservation in blood and spleen by flow cytometry, together with assessment of plasma viral titers.
